# MALDI-MSI Profiling of Effusion Cytology Cell Blocks Distinguishes High-Grade Serous Ovarian Carcinoma from Benign Effusions

**DOI:** 10.3390/cancers18142266

**Published:** 2026-07-15

**Authors:** Rita Casadonte, Alina Friemel, Oliver Klein, Stella Maren Kriegsmann, Torsten Hansen, Jörg Kriegsmann

**Affiliations:** 1 Proteopath GmbH, Max-Planck-Str. 17, 54296 Trier, Germany; 2 Department of Experimental and Clinical Medicine, University of Magna Graecia of Catanzaro, Viale Europa, 88100 Catanzaro, Italy; r.casadonte@unicz.it; 3 Department of Medicine, Faculty of Medicine and Dentistry, Danube Private University, 3500 Krems, Austria; alina.friemel@hotmail.de (A.F.); joerg.kriegsmann@dp-uni.ac.at (J.K.); 4 MVZ Einbeck, Am Kornfeld 4, 86932 Pürgen, Germany; 5 Imaging Mass Spectrometry Unit, Berlin Institute of Health at Charité—Universitätsmedizin Berlin, 13353 Berlin, Germany; oliver.klein@bih-charite.de; 6 Klinik für Dermatologie und Venerologie, Universitätsklinikum Freiburg, Hauptstraße 7, 79104 Freiburg, Germany; stella.kriegsmann@uniklinik-freiburg.de; 7 MVZ Histology Cytology and Molecular Diagnostics Trier GmbH, Max-Planck-Str. 5, 54296 Trier, Germany; torsten.hansen@patho-trier.de (T.H.);

**Keywords:** ascites, clinical diagnostics, cytology, FFPE-cells, MALDI, mass spectrometry imaging, ovarian cancer

## Abstract

**Background/Objectives:** Cytological analysis of pleural and peritoneal effusions is minimally invasive but may show limited sensitivity for malignancy detection because tumor cells can be scarce or morphologically difficult to identify. Proteomics-based mass spectrometry imaging (MSI) enables direct molecular profiling of cytological specimens and may improve the distinction between malignant and benign samples. This study investigated whether MALDI-based MSI profiling of formalin-fixed paraffin-embedded (FFPE) cytology cell blocks could discriminate high-grade serous ovarian carcinoma (HGSOC) from benign effusions and identify discriminatory peptide signatures. **Methods:** Forty-one FFPE cytological specimens derived from pleural and peritoneal effusions were analyzed, including 18 malignant samples and 23 benign control specimens with reactive or inflammatory cytological backgrounds. MALDI-MSI analyses were performed using proteomic profiling. In a preliminary comparative experiment, two section thicknesses (3 µm and 5 µm) were evaluated to optimize ion peak intensity yield. Classification analyses using linear discriminant analysis (LDA) and support vector machine (SVM) models were performed to identify discriminatory peptide signatures. **Results:** Comparative analysis of average mass spectra identified multiple differentially expressed ions with significant discriminatory performance (AUROC ≥ 0.7 or ≤0.3; Wilcoxon/Kruskal–Wallis, *p* < 0.001). Classification analyses achieved accuracy ranged from 91% to 94% for the discrimination of malignant and benign samples. Differential proteomic profiling identified Complement C3, Perilipin-3, arachidonate 5-lipoxygenase, Leukotriene A-4 hydrolase, fibrinogen beta and gamma chains, and serotransferrin as discriminatory proteins associated with immune modulation, lipid metabolism, cytoskeletal and extracellular matrix organization, and metabolic regulation. Notably, Complement C3 was found to be overexpressed in malignant tumor cells, supporting its potential role as a marker of tumor presence and progression. **Conclusions**: Proteomics-based mass spectrometry imaging enabled reliable discrimination of HGSOC from benign cytological specimens and revealed cancer-associated proteins linked to relevant biological processes. These findings support MALDI-MSI as a complementary molecular approach for the classification of challenging cytological specimens in routine diagnostic pathology.

## Simple Summary

Analysis of pleural and peritoneal body fluids is commonly used in clinical practice to detect cancer because sample collection is minimally invasive. However, conventional cytological examination has limited sensitivity because cancer cells may be scarce or difficult to recognize. In this study, we explored the use of mass spectrometry imaging, a technology that measures proteins directly within tissue sections, to improve the distinction between malignant and non-malignant samples using cytological material. With this approach we were able to accurately differentiate high-grade serous ovarian carcinoma from benign conditions and identify cancer-related protein signatures. Our findings suggest that this approach could enhance the diagnostic value of cytological specimens and support more reliable cancer detection in routine clinical settings.

## 1. Introduction

Body cavity fluids, such as pleural and peritoneal effusions, are demanding but extremely clinically useful specimens for the initial diagnosis of cancer and for the early identification of the disease recurrence in patients [1]. Diagnosing diseases by looking at single cells and small clusters of cells has attracted growing attention in the era of personalized medicine. These specimens are frequently collected in routine practice through minimally invasive procedures and often constitute the first available evidence of malignancy [2].

In ovarian cancer in particular, the predominant route of tumor spread is dissemination within the peritoneal cavity, where malignant cells readily exfoliate from the primary tumor and implant on peritoneal surfaces [3]. Pleural effusion, when present, is commonly a consequence of lymphatic dissemination from the abdomen to the pleural space. In this context, serous effusions represent not only a manifestation of advanced disease but also a valuable diagnostic resource, as cytological evaluation plays a crucial role in detecting malignant cells and supporting disease staging and clinical management [4].

Early and accurate detection is especially important in the context of ovarian cancer, which is commonly diagnosed at an advanced stage due to the absence of specific early symptoms and effective population-based screening strategies [5].

Conventional cytological evaluation of effusion specimens relies on morphological assessment complemented by immunocytochemistry. While highly informative, this approach can be limited by subjective interpretation, overlapping cytomorphologic features between reactive and malignant cells, and insufficient cellularity in some samples [6,7]. Moreover, cytology does not routinely provide detailed molecular information that may be relevant for diagnosis, prognosis, and therapeutic stratification. As precision oncology continues to evolve, there is an increasing need for analytical techniques capable of extracting comprehensive molecular signatures directly from routinely collected clinical specimens.

Mass spectrometry imaging (MSI) has emerged as a robust platform for spatially resolved molecular characterization, enabling label-free detection and mapping of protein distributions directly within clinical specimens [8]. By preserving cellular and tissue architecture while generating high-dimensional proteomic datasets, this approach provides detailed insight into the molecular composition of samples in situ. Such spatially resolved proteomic profiling can improve diagnostic precision and facilitate the identification of disease-specific protein signatures [9,10]. To date, the majority of clinical and translational studies employing MSI have focused on FFPE or fresh-frozen tissue sections, where spatially resolved proteomic profiling has demonstrated value for tumor classification, biomarker discovery, and outcome prediction [11,12,13,14,15]. Recent reviews emphasize that the clinical adoption of MALDI-MSI increasingly depends on standardized preanalytical workflows, reproducible data processing, robust machine learning validation, and transparent reporting [16,17]. Optimized sample preparation methods and high-resolution mass spectrometry imaging techniques have been developed to facilitate low-cell and single-cell analyses [18,19]. Emerging studies have demonstrated successful protein and peptide profiling in cultured cancer cell lines [20], three-dimensional spheroid models [21], and cytological preparations, including liquid-based cytology slides and cell block material [22]. These developments suggest that MSI is technically feasible in minimally invasive specimens and supports its extension to body cavity fluid-derived cytology samples.

When applied to cytological specimens derived from pleural and peritoneal effusions, MSI has the potential to complement morphology-based evaluation by providing objective molecular information directly from routinely processed FFPE cell blocks. This is particularly relevant because recent translational studies describe ovarian cancer ascites as a biologically active ecosystem that contributes to peritoneal dissemination, immune suppression, metabolic adaptation, and therapy resistance [3,4]. In the present study, we investigated whether MALDI-MSI-based proteomic profiling could distinguish HGSOC from benign pleural and peritoneal effusions and identify discriminatory molecular signatures associated with malignant transformation.

## 2. Materials and Methods

### 2.1. Sample Collection

Samples were used from pleural and peritoneal body fluid specimens. Forty-one cases were selected based on the presence of malignancy on the pleural effusions (*n* = 7) and ascites (*n* = 11), and benign pleural effusion specimens (*n* = 23). Additional clinical information about the study population is reported in Supplementary Table S1. Samples were mixed with ethanol 50% and concentrated by centrifugation at 1200 rpm for 10 min. Cell pellets were then fixed in 4% buffered formalin, placed inside a histological cassette and run through a standard, automated tissue processor (Sakura Tissue-TEK-Xpress-X120, Sakura Finetek Europe B.V., Alphen aan den Rijn, The Netherlands) for cell block preparation. In a preliminary comparative experiment two different tissue section thicknesses (3, 5 µm) were tested on tumor and non-tumor samples to evaluate the ion peak intensity yield. Sections of each paraffin block were mounted either onto regular glass slides for hematoxylin and eosin staining or onto indium-tin oxide (ITO)-coated glass slides (Bruker Daltonik, Bremen, Germany) for MALDI mass spectrometry imaging analysis.

### 2.2. Sample Preparation and MSI Analysis

The workflow for mass spectrometry imaging analysis of FFPE sections required some preanalytical steps. These included: (1) dewaxing in xylene 100% (2 × 5 min each); (2) re-hydration into a series of graded alcohols baths (isopropanol 100%, ethanol 100%, ethanol 95%, ethanol 70%, and ethanol 50%), and water (2 × 3 min each); (3) epitope retrieval in 10 mM Tris buffer pH = 9 at 95 °C (20 min); (4) on-tissue digestion by application of 16 layers of trypsin solution (0.025 µg/µL trypsin in 20 mM ammonium bicarbonate) using a reagent sprayer (TM-Sprayer, HTX-Technologies, Chapel Hill, NC, USA, and incubation at 50 °C for 2.5 h; (5) on-tissue peptide crystallization by spraying 4 layers of α-cyano-4-hydroxycinnamic acid (CHCA) matrix solution (10 mg/mL in 70% acetonitrile, 1% trifluoroacetic acid) with the same reagent devise used before. The optimized parameter setting for the trypsin and matrix spraying method is described in Supplementary Table S2.

Mass spectrometry imaging data was performed using a rapifleX MALDI Tissue-typer mass spectrometer (Bruker Daltonik, Bremen, Germany) in positive-ion reflector mode with a laser frequency of 10 kHz. Then, 200 spectra were averaged at each tissue spot position with single Smartbeam Parameter at 20 µm × 20 µm scan range, in the *m*/*z* range 500–3200. The acquisition method was externally calibrated with a mixture of standard peptides, including bradykinin fragment 1–7, angiotensin II, angiotensin I, sub-stance P, bombesin, renin substrate, ACTH clip 1–17, ACTH clip 18–39, somatostatin 28 (Bruker Daltonik, peptide calibration standard II, Part No:8222570, Bremen, Germany). Ion images were assembled and visualized using FlexImaging (version 5.0) or SCiLS Lab (version 2023a 11.00.14179) (Bruker Daltonik) software packages. After MALDI acquisition, the matrix was removed in 2 × 100% ethanol baths (3 min each), then the samples were stained with Hematoxylin and Eosin (HE). Slides were scanned with a 40× objective magnification using the Aperio AT2 automated slide scanner Leica Biosystems, Wetzlar, Germany). Regions of interest (ROIs) were manually annotated by a board-certified pathologist (JK) based on the corresponding high-resolution digitized HE-stained tissue sections. The diagnostic classification of the cases was established as part of the routine diagnostic work-up and was supported, where appropriate, by ancillary immunocytochemical/immunohistochemical studies. Thus, the annotated tumor regions were based on comprehensive morphologic assessment in conjunction with the available ancillary diagnostic findings. Given the extensive expertise of the annotating pathologist and the integration of routine diagnostic and immunocytochemical findings, no additional multi-observer review was performed. The annotations were subsequently transferred to the corresponding MALDI-MSI datasets for spectral extraction and analysis.

### 2.3. Data Processing and Classification Analysis

Mass spectral data was baseline subtracted during data acquisition using TopHat algorithm and then preprocessed in SCiLS Lab (version 2023a 11.00.14179) software (Bruker Daltonik) and R (version 3.5.3; https://www.r-project.org) for spectra recalibration. Peak picking was performed using the orthogonal matching pursuit algorithm to determine relevant peaks. Mass spectra from tumor annotated regions of both benign and malignant samples were imported in R (version 3.5.3 and 4.0.3; https://www.r-project.org) undergoing to a pipeline procedure described by Boskamp et al. [23]. Briefly, spectra were normalized with intensity profile normalization (IPN), resampled by binning all spectral intensities over a peak-width of 0.4 Da in the *m*/*z* range from 700 Da to 2500 Da, and smoothed with “gaussian” spatial smoothing algorithm (Gaussian kernel inside a radius of 105 µm with a sigma of 75 µm).

To discriminate HGSOC from benign samples, predictive models were created using LDA and SVM algorithms and assessed by leave-one-out cross-validation (LOOCV). Classification analysis was performed considering individual spectra and mean spectra from individual patients. Because of the limited cohort size (*n* = 41 patients), the dataset was not divided into separate training and validation cohorts. Instead, LOOCV was used, whereby each spectra/patient was iteratively excluded from model generation and used as an independent test case. To minimize potential bias caused by spectral similarity within individual patient samples, cross-validation was performed at the patient level whenever patient-mean spectra were used, ensuring that spectra derived from the same patient were excluded simultaneously from the training dataset during validation. A selected list of *m*/*z* peptide ions was statistically created based on AUROC analysis or Stepwise Forward Feature Selection (FFS) and used by LDA and SVM models for classification analysis.

### 2.4. Protein Identification

Serial tissue sections from randomly selected high-grade serous ovarian carcinoma and non-tumor samples were subjected to bottom–up proteomic characterization to assign the peptide features detected by MALDI-MSI. Proteomic analysis was performed using a nano-LC-ESI-MS/MS, and a timsTOF platform (Bruker Daltonik). Tissue preparation followed the same protocol adopted for MALDI-MSI through the on-tissue enzymatic digestion stage [12]. Peptides generated during digestion were extracted by applying 100 µL of 0.1% TFA at room temperature. The peptide extracts were purified using C18 ZipTip pipette tips (Merck KGaA, Darmstadt, Germany), vacuum-dried, and reconstituted in 20 µL of 0.1% TFA before storage at −20 °C pending MS/MS analysis. A total of 6 µL of peptide extract was analyzed via nanoHPLC (Vanquish UHPLC systems, Thermo Fisher Scientific, Germering, Germany) connected to an ESI-timsTOF-MS (timsTOF HT Flex, Bruker Daltonik) by a captive spray source II (Bruker Daltonik) in a data-dependent acquisition (DDA) and parallel accumulation–serial fragmentation (PASEF) mode. The derived MS/MS spectra were searched against the human Swiss-Prot database using FragPipe (FragPipe version 21.1, including MSFragger version 4.0, IonQuant version 1.10.12 and Philosopher version 5.1.0 [24]. Default settings were used. Trypsin was specified as the proteolytic enzyme, allowing up to two missed cleavages. Protein assignments of MALDI-MSI *m*/*z* features were performed by comparison with peptides identified by LC-MS/MS. Candidate protein identifications required the detection of more than one matching peptide and a mass difference of less than 0.15 Da between the MALDI-MSI and LC-MS/MS *m*/*z* values. Mass differences up to 0.5 Da were also considered acceptable, consistent with previously reported approaches employing thresholds of up to 1 Da. When multiple peptide candidates exhibited comparable mass differences, the peptide with the highest identification confidence was selected based on the lowest expectation value (E-value), typically requiring an E-value < 0.01.

Unique peptide sequences were not required for protein assignment, as biologically relevant proteins may also be represented by shared peptide sequences. To support protein annotation, the spatial distributions of candidate peptides were evaluated, and only peptides exhibiting similar localization patterns (co-localization) within the tissue sections were considered supportive of the same protein assignment. Post-translational modifications were generally not considered during the matching process. However, when a protein assignment relied on only two peptides, additional verification was performed to ensure that modified and unmodified peptide variants corresponded to the same peptide sequence; otherwise, the protein assignment was excluded.

## 3. Results

**Figure 1:**
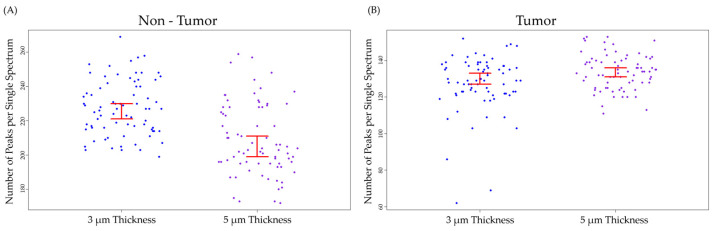
Spectral data comparison between two different tissue thicknesses (3, 5 µm) of non-tumor (**A**) and tumor (**B**) samples. The median peak count was higher in the 3 µm compared the 5 µm thickness in the non-tumor sample (**A**), but not significantly different between the two thickness in the tumor (**B**).

Preliminary analyses were performed to determine the optimal section thickness from the cell-FFPE block. Spectral data from cell blocks were analyzed to determine the optimal section thickness. Visual inspection and comparison of the spectra revealed that, in non-tumor samples, the 3 µm sections exhibited a higher overall number of detected *m*/*z* peaks (median = 309) within the 600–3000 *m*/*z* range compared to 5 µm sections (median = 275). In tumor samples, however, the median peak counts were similar between 3 µm (median = 219) and 5 µm (median = 224) sections (Figure 1). Additionally, peak intensities across nearly all *m*/*z* species were lower in the thicker tissue sections for both tumor and non-tumor samples (Supplementary Figure S1). The comparison of 3 µm and 5 µm sections was performed as a methodological optimization using a limited number of representative HGSC and benign specimens. Based on the consistently higher peak counts and improved signal quality observed in the 3 µm sections, this thickness was selected for all subsequent analyses.

### 3.1. Classification Analysis

The classification models were generated using spectra extracted from manually annotated regions of interest. A total of 5376 spectra from 41 patients were included, comprising 2759 spectra from 18 HGSC cases and 2617 spectra from 23 non-tumor cases. The median number of spectra per patient was 148 (interquartile range (IQR): 83–184) for HGSC and 95 (IQR: 49–151) for non-tumor samples, according to the size and quality of the annotated regions of interest. To assess the potential impact of patient-related spectral dependencies on classification performance, additional analyses were performed at the patient level. Leave-one-out cross-validation (LOOCV) was performed at both the spectrum and patient levels. In the spectrum-level analysis, one spectrum was excluded at each iteration and classified using a model generated from the remaining spectra. In the patient-level analysis, all spectra from one patient were excluded and classified using a model generated from the remaining patients. This approach ensured that no spectra from the same patient were simultaneously included in both the training and testing phases.

**Figure 2:**
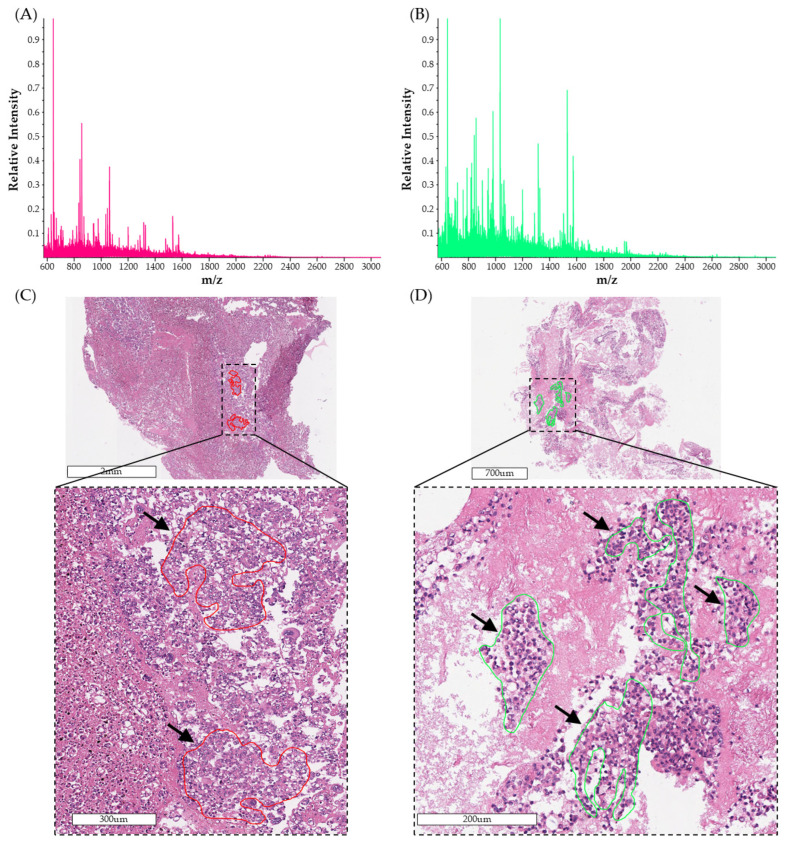
Representative average MALDI-MSI spectra obtained from HGSOC (**A**) and benign cytological specimens (**B**). Representative hematoxylin and eosin (H&E)-stained sections with annotated regions indicated by black arrows are shown for cancer (**C**) and non-cancer (**D**).

Comparative analysis of the average peptide spectra revealed clear differences between high-grade serous ovarian carcinoma and non-cancer cells (Figure 2).

The resulting mass spectra yielded 482 peaks of various intensity over an intensity range of one to two orders of magnitude in the *m*/*z* range 500–3000. To identify the most discriminatory features, peaks were filtered based on Wilcoxon statistical analysis (*p* < 0.001) together with AUROC values ≥ 0.7 or ≤0.3. Peaks with AUROC values below 0.5 were considered discriminatory for the benign phenotype, whereas peaks above 0.5 were associated with tumor spectra. This approach resulted in the selection of 21 discriminant *m*/*z* peaks capable of distinguishing tumor from benign spectra with high accuracy that were subsequently used to generate an LDA classification model (Table 1). Log2 fold change analysis was performed using normalized peak intensities calculated as log2(benign/tumor). Negative Log2FC values therefore indicated higher abundance in tumor tissue, whereas positive values reflected enrichment in benign specimens (Table 1).

Classification analyses were performed using two independent software platforms, SCiLS Lab and R, to evaluate the robustness of the predictive models. In both approaches, Linear Discriminant Analysis (LDA) was applied, while Support Vector Machine (SVM) classification was additionally implemented in the R statistical environment. All models were used with leave-one-out cross-validation (LOOCV). Overall, the results obtained from the two software platforms were highly comparable, demonstrating consistent discriminatory performance between tumor and non-tumor tissue spectra.

In the classification at the spectra-level using SCiLS Lab, the LDA model achieved an overall classification accuracy of 93%. A total of 2638 out of 2759 tumor spectra were correctly classified, corresponding to a sensitivity of 96%, whereas 2384 out of 2617 non-tumor spectra were correctly assigned, corresponding to a specificity of 91%.

Similarly, in R, the best classification performance using the LDA algorithm was obtained when all individual spectra were included and six discriminative features selected through Stepwise Forward Feature Selection (FFS) analysis were applied. Under these conditions, tumor spectra were correctly classified with an accuracy of 93%. In the same classification setting, the SVM model demonstrated a slightly improved discriminative capability, correctly classifying tumor spectra with an accuracy of 94% (Table 2).

In the classification at patient-level, the overall classification accuracy remained high, yielding accuracies of 80% (using SCiLS Lab software) and 88% (using R software) for LDA model and 93% (using R software) for SVM model, demonstrating that the discriminatory performance was maintained when classification was assessed at the patient level (Table 2). The robustness of the patient-level classification results was further assessed by calculating 95% confidence intervals for sensitivity, specificity, precision and overall accuracy. Balanced accuracy was additionally calculated to account for potential differences in class sizes by averaging sensitivity and specificity (Supplementary Table S3). The highest performance was achieved using the SVM classifier, yielding a sensitivity of 94.4% (95% CI: 74.2–99%), specificity of 91.3% (95% CI: 73.2–97.6%), and overall accuracy of 92.7% (95% CI: 80.6–97.5%) (Supplementary Table S3). Although the confidence intervals are relatively wide due to the limited cohort size, the results support the ability of MALDI-MSI-based classification to discriminate HGSC from benign effusions at the patient level.

**Figure 3:**
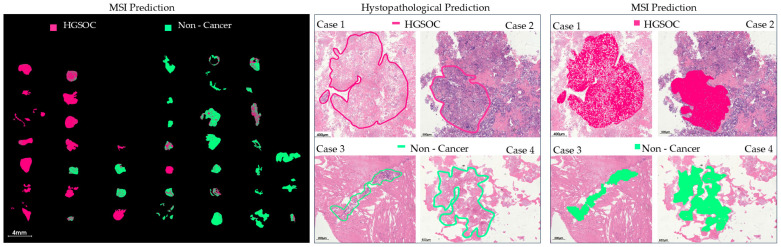
MALDI-MSI-based classification maps generated using the LDA model. High-grade serous ovarian carcinoma (HGSOC) regions are shown in magenta and benign regions in green. Corresponding H&E-stained sections and MSI-based predictions are displayed.

The results of the LDA classification model were visualized using a color-encoded representation, allowing intuitive discrimination between the different phenotypes. Distinct colors were assigned to each class, with magenta representing high-grade serous ovarian carcinoma spectra and green representing non-tumor spectra (Figure 3). This visualization clearly highlighted the spatial distribution and separation of the two phenotypic groups, further supporting the strong discriminatory performance of the proteomic classification model. These results demonstrate the robustness of the proteomic signature and highlight the ability of the MALDI-MSI-based approach to accurately distinguish malignant from non-malignant cytological specimens.

Among the 21-discriminant *m*/*z* peaks, some *m*/*z* ion peaks were significantly overexpressed in tumor samples (*m*/*z* 766.383, 818.4, 819.425, 871.486), whereas others showed reduced intensity compared to non-tumor samples (*m*/*z* 981.48, 823.452, 1034.551, 1184.583, 760.408, 1055.566, 1608.758, 1193.55, 886.43, 1056.517, 1283.612, 1.683.956, 1195.566, 1684.953, 1897.911, 1916.949, 1917.901), supporting the presence of distinct proteomic signatures associated with the malignant phenotype (Table 1.).

### 3.2. Protein Identification

The most relevant discriminatory features were subjected to MS/MS-based identification to determine their molecular identity. MALDI MSI and LC–MS/MS discriminant *m*/*z* values were further cross-compared with protein identification, applying a stringent criterion requiring at least two matching peptides and a mass error < 1 ppm. Although four discriminant ion peptides were initially found to be highly expressed in tumor samples, subsequent MS/MS analysis revealed that only one (*m*/*z* 871.486), identified as Complement C3, retained a concordant tumor-associated increase at the protein level. This observation likely reflects the complexity of peptide-to-protein assignment and the heterogeneous contribution of individual peptide species to the detected molecular signatures.

The remaining proteins identified in this study were primarily associated with peptide ions showing reduced abundance in HGSC relative to benign samples and were interpreted accordingly. *m*/*z* values showing reduced expression in tumor tissues were identified as arachidonate 5-lipoxygenase (*m*/*z* 981.48), CD44 antigen (*m*/*z* 1184.583), endoplasmic reticulum resident protein 29 (*m*/*z* 1608.758), fibrinogen beta chain (*m*/*z* 1684.953), fibrinogen gamma chain (*m*/*z* 1034.551), heat shock protein HSP 90-beta (*m*/*z* 886.43), perilipin-3 (*m*/*z* 1193.55); serotransferrin (*m*/*z* 1195.566, 1283.612), thymidine phosphorylase (*m*/*z* 1055.566), phosphoglycerate mutase 1 (*m*/*z* 1683.956), SH3 domain-binding glutamic acid-rich-like protein 3 (*m*/*z* 1056.517), Y-box-binding protein 1 (*m*/*z* 1897.911), and zyxin (*m*/*z* 1917.901). The detailed outcomes of the identification workflow are provided in Supplementary Table S4.

## 4. Discussion

In the present study, we established a MALDI-based proteomic workflow on FFPE-derived cytology cell blocks to discriminate HGSOC from benign effusions and to characterize associated molecular alterations. A key preliminary finding was that section thickness influenced spectral quality, with 3 µm sections yielding a higher number of detectable *m*/*z* features and improved signal intensity compared to 5 µm sections, particularly in benign samples. This observation is consistent with previous reports indicating that thinner FFPE sections can enhance matrix penetration and ionization efficiency, thereby improving peptide detection sensitivity in MSI [25,26]. Recent reviews of MALDI-MSI implementation further emphasize that standardized sample preparation and reproducible computational pipelines are key prerequisites for clinical translation [17,22]. The reduced signal intensity observed in thicker sections likely reflects ion suppression effects and incomplete analyte extraction, reinforcing the importance of standardized preanalytical conditions for reproducible MSI analyses.

The classification analysis demonstrated that MALDI-derived peptide signatures can robustly distinguish HGSOC from benign effusion-derived cell blocks. From a total of 482 detected peaks in the 500–3000 *m*/*z* range, a subset of 21 discriminant peaks (AUROC ≥ 0.7 or ≤0.3; *p* < 0.001) was sufficient to generate an accurate classification model. The classification accuracy achieved at the spectrum-level in the present study (91–94%) is particularly noteworthy in the context of routine effusion cytopathology. Conventional cytological evaluation of pleural and peritoneal effusions, although highly specific, may show variable sensitivity depending on tumor cell content, sampling quality, and observer experience. Reported sensitivities for routine cytology in malignant effusions commonly range between approximately 60–85%, particularly in ovarian cancer-associated serous effusions where reactive mesothelial cells and inflammatory backgrounds may complicate interpretation [2,6]. Ancillary immunocytochemical approaches and cell block preparations can substantially improve diagnostic accuracy; however, these methods remain dependent on sufficient cellularity, antibody selection, and morphological interpretation. In contrast, the MALDI-MSI-based workflow presented here provides objective molecular classification directly from routinely processed FFPE cytology cell blocks while preserving tissue architecture. Nonetheless, the classification performance remained robust when evaluated at the patient level using LOOCV, suggesting that the observed discrimination was not solely driven by the large number of spectra acquired from individual specimens. The high classification performance observed in the present study therefore suggests that MSI-based proteomic profiling may represent a valuable complementary diagnostic approach, particularly in diagnostically challenging specimens with limited or equivocal cytomorphological findings.

Importantly, the achieved classification accuracy is also comparable to or exceeds several previously reported MSI-based tumor classification studies performed on solid tissue specimens, further supporting the robustness and translational potential of the present workflow for minimally invasive cytological diagnostics [7]. These results align with previous studies showing that limited panels of proteomic features can achieve high classification accuracy in solid tumors [27]. They are also consistent with recent MALDI-MSI work in HGSOC, where spatial proteomic profiling identified proteins associated with chemotherapy resistance and clinical outcome, supporting the broader translational value of MALDI-MSI in ovarian cancer research [28].

Beyond classification, we identified a distinct proteomic signature differentiating HGSOC from benign effusion-derived cells, characterized by alterations in complement activation, lipid metabolism, extracellular matrix organization, tumor-associated inflammation, and cytoskeletal organization. These findings are consistent with established hallmarks of cancer, including tumor-promoting inflammation and metabolic reprogramming [29]. A prominent finding was the upregulation of complement component C3 in tumor samples. Recent work has strengthened the biological plausibility of this observation by showing that adipocyte-cancer cell interactions in ovarian cancer can induce lipid transfer, activate integrated stress response signaling, and upregulate complement proteins including C3 and C5; C3 inhibition reduced invasive features and tumor progression in experimental models [30]. In ovarian cancer, elevated C3 expression has also been reported in tumor tissues and ascitic fluid, where it has been associated with disease progression and adverse clinical outcome [31]. Our findings therefore support complement activation as a relevant component of the proteomic phenotype of malignant effusions, while causal conclusions require independent functional validation.

Conversely, several proteins involved in cytoskeletal organization and cell adhesion were reduced in tumor specimens. Zyxin, a focal adhesion-associated protein involved in actin remodeling and mechano-transduction, has been described as a regulator of cell adhesion and migration. Loss or reduced expression of zyxin has been associated in several malignancies with altered focal adhesion dynamics and increased cellular plasticity, potentially facilitating tumor invasion and metastatic dissemination. Its decreased abundance in the present study may therefore reflect disruption of normal cytoskeletal architecture during ovarian tumor progression [32].

The observed decrease in fibrinogen beta and gamma chains is consistent with a previous study that correlated fibrinogen low expression with alterations in extracellular matrix remodeling and coagulation-related signaling within the tumor microenvironment [33]. Although elevated circulating fibrinogen levels are frequently associated with ovarian cancer progression and poor prognosis, our results may show reduced fibrinogen-chain abundance within tumor-cell-enriched regions due to loss of normal stromal architecture and changes in vascular or inflammatory composition. These findings therefore likely reflect local microenvironmental remodeling rather than systemic coagulation activity [33].

The decrease in serotransferrin expression has been suggested as perturbation of iron metabolism in ovarian tumors [34]. Cancer cells frequently reprogram iron homeostasis to support proliferation and oxidative metabolism. Altered transferrin-related pathways have been associated with aggressive ovarian cancer phenotypes and metabolic adaptation under hypoxic conditions [34]. Reduced levels of these transport proteins may reflect a shift toward intracellular iron retention, favoring tumor growth while increasing oxidative stress [35].

The downregulation of Perilipin-3 (PLIN3), arachidonate 5-lipoxygenase (ALOX5), and Leukotriene A-4 hydrolase (LTA4H) in malignant pleural and peritoneal effusions has been linked to cellular lipid metabolism, lipid storage, and inflammatory signaling [36]. Recent proteomic analyses of ovarian cancer ascites further support the concept that malignant ascitic fluid reflects disease-associated metabolic remodeling, including changes in nutrient utilization, cholesterol-related pathways, and immunoregulatory signaling during disease progression [37]. Perilipin-3 is a lipid droplet-associated protein involved in intracellular lipid storage and metabolism. Its reduced abundance may therefore reflect altered lipid handling in effusion-derived tumor cells, consistent with the known role of lipid metabolic reprogramming in ovarian cancer progression [38].

The observed reduction of arachidonate 5-lipoxygenase further supports deregulation of lipid-mediated inflammatory pathways. This enzyme participates in leukotriene biosynthesis and inflammatory signaling, processes frequently implicated in tumor progression and immune modulation. Its decreased abundance is consistent with a study indicating selective rewiring of arachidonic acid metabolism in the analyzed tumors [39]. Leukotriene A-4 hydrolase (LTA4H), an enzyme involved in leukotriene B4 biosynthesis and inflammatory lipid signaling [36,40], was downregulated in ovarian tumor tissues. Previous studies have associated altered arachidonic acid metabolism with immune modulation and metabolic adaptation in ovarian cancer. Accordingly, the concurrent reduction of ALOX5 and LTA4H observed in our cohort may be indicative of changes in leukotriene-mediated inflammatory signaling within the tumor microenvironment. However, the precise biological implications of these findings require further investigation [37].

Additional downregulated protein included Endoplasmic reticulum resident protein 29 (ERp29) which was found to be involved in protein folding, endoplasmic reticulum stress response, and epithelial cell differentiation [41]. In several cancers reduced ERp29 expression has been associated with loss of epithelial differentiation, enhanced invasiveness and metastatic potential [42]. Therefore, observing lower ERp29 expression in tumor vs non-tumor tissue is biologically plausible and consistent with tumor progression mechanisms.

The observed downregulation of heat shock protein HSP90-beta and CD44 was unexpected because both proteins have been described as oncogenic markers with overexpression in primary tumors [43,44]. This finding should be interpreted cautiously and may reflect biological differences between primary tumor tissue and exfoliated tumor cells within pleural and peritoneal effusions. Lower CD44 abundance may be associated with altered cell adhesion and spheroid biology in ascitic environments, whereas changes in chaperone abundance may reflect context-dependent stress adaptation rather than a universal tumor-promoting mechanism. These observations require validation in larger cohorts and orthogonal assays [45].

Interestingly, several proteins identified as downregulated in the present study, including phosphoglycerate mutase 1 (PGAM1, *m*/*z* 1683.956), SH3 domain-binding glutamic acid-rich-like protein 3 (SH3BGRL3, *m*/*z* 1056.517), and Y-box-binding protein 1 (YB-1, *m*/*z* 1897.911), have previously been associated with tumor progression and, in some cases, reported as overexpressed in ovarian or other epithelial cancers [46,47]. However, these apparent discrepancies do not necessarily invalidate the present findings and may instead reflect the biological heterogeneity of ovarian tumors, differences in sample type, tumor microenvironment, or the distinct spatially resolved proteomic approach employed in the present study. Therefore, the reduced expression observed in the present study may represent context-dependent metabolic and microenvironmental adaptations characteristic of the analyzed tumor specimens.

Overall, the proteins identified in this study point to key biological processes involved in HGSC pathogenesis, rather than definitive proof of altered protein expression. While individual protein functions are discussed above, Supplementary Table S5 summarizes the main proteins distinguishing HGSC from benign ovarian tissues, their potential direction of regulation, and biological relevance. This provides a concise overview that may aid clinicians and pathologists in interpreting the proteomic alterations in the context of ovarian cancer progression.

Compared with standard immunocytochemistry (ICC), the proteomic approach used in this study provides a broader, unbiased assessment of protein alterations without requiring predefined antibodies. While ICC remains a well-established, cost-effective, and widely accessible technique in routine pathology for validating specific protein targets, it is inherently limited to a small number of markers per assay. In contrast, MALDI-MSI and LC-MS/MS-based workflows enable simultaneous evaluation of multiple proteins and their spatial distribution within tissue sections, offering a more comprehensive molecular characterization of tumor heterogeneity. Nevertheless, clinical implementation is currently limited by higher costs, instrument complexity, and the need for specialized expertise, although ongoing technological advances are expected to improve feasibility in clinical settings.

## 5. Conclusions

This study demonstrates that MALDI-based proteomic profiling of FFPE-derived cytology cell blocks can effectively distinguish high-grade serous ovarian carcinoma from benign pleural and peritoneal effusions. A panel of discriminatory peptide features enabled robust classification performance across independent analytical approaches.

The identified proteomic alterations were associated with pathways involved in complement activation, lipid metabolism, extracellular matrix remodeling, inflammatory signaling, and metabolic regulation, supporting the biological relevance of the detected molecular signatures.

Several limitations should be acknowledged. First, the study cohort was relatively small and consisted exclusively of high-grade serous ovarian carcinoma cases. Therefore, the applicability of the identified proteomic signatures to other ovarian cancer histological subtypes remains to be determined. The present study was designed as a proof-of-concept investigation using well-characterized HGSC and benign effusions with a high degree of diagnostic confidence to establish robust MALDI-MSI-based classification models. Consequently, diagnostic challenging or equivocal cases were not specifically included in the study cohort. Future studies, including a broader spectrum of benign and malignant effusions as well as diagnostic challenging effusions will be necessary to fully establish the clinical specificity and diagnostic utility of this method. Second, although classification performance was high, independent external validation in larger multi-center cohorts will be required before clinical implementation. Finally, the present study was designed to evaluate molecular classification performance and does not establish causal mechanistic relationships between the identified proteins and ovarian cancer progression.

Importantly, these findings highlight the potential of MALDI-MSI as a complementary molecular tool for the evaluation of challenging cytological specimens in routine diagnostic pathology. Future studies should focus on validation in larger independent cohorts and on assessing reproducibility and clinical applicability.

## Figures and Tables

**Table 1:** Discriminant *m*/*z* features for LDA classification model.

*m*/*z*	Wilcoxon *p*-Value	AUROC Tumor/Non-Tumor *	Average Intensity (Peak Maximum)—Non Tumor—Total Ion Count	Average Intensity (Peak Maximum)—Tumor—Total Ion Count	Log2 Fold Change NT/T
760.408	<0.001	0.2	11.0073	16.4256	−0.5775
766.383	<0.001	0.72	17.002	22.1080	−0.3789
818.4	<0.001	0.75	12.3358	17.4900	−0.5037
819.425	<0.001	0.74	17.5731	9.9600	0.8192
823.452	<0.001	0.18	8.9250	12.4805	−0.4838
871.486	<0.001	0.75	21.2571	8.5071	1.3212
886.43	<0.001	0.2	34.8579	14.7672	1.2391
981.48	<0.001	0.15	12.6703	8.3263	0.6057
1034.551	<0.001	0.19	38.8434	14.2133	1.4504
1055.566	<0.001	0.2	12.7696	6.7956	0.91
1056.517	<0.001	0.22	10.4609	6.3430	0.7218
1184.583	<0.001	0.19	9.2161	5.4898	0.7474
1193.55	<0.001	0.2	14.8860	6.3130	1.2376
1195.566	<0.001	0.24	8.5890	5.4511	0.6559
1283.612	<0.001	0.22	7.1594	4.6473	0.6234
1608.758	<0.001	0.2	6.9179	3.5336	0.9692
1.683.956	<0.001	0.23	7.7766	3.5447	1.1335
1684.953	<0.001	0.24	5.2168	3.0841	0.7583
1897.911	<0.001	0.27	3.1501	2.4719	0.3498
1916.949	<0.001	0.29	3.0274	2.3162	0.3863
1917.901	<0.001	0.3	2.8022	2.2919	0.29

* AUROC = Area under the receiver Operating characteristics. AUROC was calculated using tumor samples as the positive class and non-tumor samples as the negative class.

**Table 2:** MSI-Based Proteomics Classification Results.

Classification Strategy	Model	Cross Validation	Phenotype	Total N. Spectra	True	False	Correct Calssify %	Classification Accuracy %
Spectrum-Level	LDA ^1^—SCiLS Lab	LOOCV ^2^	Tumor	2759	2638	121	96	93
			Non-tumor	2617	2384	233	91	
	LDA—R Statistics	LOOCV	Tumor	2759	2661	98	96	93
			Non-tumor	2617	2349	268	90	
	SVM ^3^—R Statistics	LOOCV	Tumor	2759	2624	135	95	94
			Non-tumor	2617	2412	205	92	
**Classification Strategy**	**Model**	**Cross Validation**	**Phenotype**	**Total N. Patient**	**True**	**False**	**Correct Calssify %**	**Classification Accuracy %**
Patient-Level	LDA—SCiLS Lab	LOOCV	Tumor	18	15	3	83	80
			Non-tumor	23	18	5	78	
	LDA—R Statistics	LOOCV	Tumor	18	16	2	87	88
			Non-tumor	23	20	3	89	
	SVM—R Statistics	LOOCV	Tumor	18	17	1	94	93
			Non-tumor	23	21	2	91	

^1^ LDA = Linear discriminant analysis. ^2^ Loocv = leave-one-out cross-validation. ^3^ SVM = Support Vector Machine.

## Data Availability

The data presented in this study are available from the corresponding author upon reasonable request.

## Supplementary Materials

The following supporting information can be downloaded at https://www.mdpi.com/article/10.3390/cancers18142266/s1, Figure S1: Spectral data comparison between two different tissue thicknesses (3, 5 µm) of non-tumor (A) and tumor (B) samples; Table S1: Clinical characteristics of the study cohort included in the study; Table S2: Setup trypsin and matrix deposition method; Table S3: Performance metrics for patient-level classification models; Table S4: Detailed MS/MS Identification Results for Annotated Peptides; Table S5: Summary of proteins differentially expressed between high-grade serous ovarian carcinoma (HGSC) and benign ovarian tissues.

## Abbreviations

The following abbreviations are used in this manuscript:

ALOX5	Arachidonate 5-lipoxygenase
AUROC	Area under the receiver operating characteristic curve
CHCA	alpha-cyano-4-hydroxycinnamic acid
DDA	Data-dependent acquisition
FFPE	Formalin-fixed paraffin-embedded
FFS	Stepwise Forward Feature Selection
H&E	Hematoxylin and eosin
HGSOC	High-grade serous ovarian carcinoma
ITO	Indium-tin oxide
LC-ESI-MS/MS	Liquid chromatography electrospray ionization tandem mass spectrometry
LDA	Linear discriminant analysis
LOOCV	Leave-one-out cross-validation
LTA4H	Leukotriene A-4 hydrolase
MALDI-MSI	Matrix-assisted laser desorption/ionization mass spectrometry imaging
MSI	Mass spectrometry imaging
PLIN3	Perilipin-3
SVM	Support vector machine

## References

[1] Dipper A., Maskell N., Bibby A. (2021). Ancillary Diagnostic Investigations in Malignant Pleural Mesothelioma. Cancers.

[2] Pinto D., Chandra A., Crothers B.A., Kurtycz D.F.I., Schmitt F. (2020). The international system for reporting serous fluid cytopathology-diagnostic categories and clinical management. J. Am. Soc. Cytopathol..

[3] Boo K.H., Lee G., Song M. (2025). Malignant ascites in ovarian cancer: New advances and translational opportunities. Transl. Oncol..

[4] Dogra S., Adhikari L., Benbrook D.M., Bohn J.A., Burgett A., Chandra V., Dockery L., Singh A., McNally L., Rai R. (2025). Harnessing ovarian cancer ascites for translational science: Models, biomarkers, and therapeutics. Mol. Cancer.

[5] Jayson G.C., Kohn E.C., Kitchener H.C., Ledermann J.A. (2014). Ovarian cancer. Lancet.

[6] Wang M., Chandra A., Cai G. (2023). The International System for Reporting Serous Fluid Cytopathology—An Updated Review. J. Clin. Transl. Pathol..

[7] Kapoor S., Samanta S., Kaur K. (2024). Role of Ancillary Techniques in Reporting Serous Fluid Cytology—“Redefining Categories, Refining Diagnosis”. J. Cytol..

[8] Kriegsmann J., Kriegsmann M., Casadonte R. (2015). MALDI TOF imaging mass spectrometry in clinical pathology: A valuable tool for cancer diagnostics (review). Int. J. Oncol..

[9] Bartusik-Aebisher D., Justin Raj D.R., Aebisher D. (2025). Detection of Protein and Metabolites in Cancer Analyses by MALDI 2000-2025. Cancers.

[10] Casadonte R., Kriegsmann M., Deininger S.O., Amann K., Paape R., Belau E., Suckau D., Fuchser J., Beckmann J., Becker M. (2015). Imaging mass spectrometry analysis of renal amyloidosis biopsies reveals protein co-localization with amyloid deposits. Anal. Bioanal. Chem..

[11] Bollwein C., Patterson N.H., Ly A., Schwamborn K., O’Sullivan N., Gonçalves J.P.L. (2025). Classification of small blue round cell tumors by integrating peptide and N-glycan mass imaging spectrometric profiles. Anal. Chim. Acta.

[12] Grzeski M., Jensen P.M., Hempel B.F., Thiele H., Lellmann J., Schallenberg S., Budach V., Keilholz U., Tinhofer I., Klein O. (2025). Integrating MALDI-MSI-Based Spatial Proteomics and Machine Learning to Predict Chemoradiotherapy Outcomes in Head and Neck Cancer. Int. J. Mol. Sci..

[13] Casadonte R., Kriegsmann M., Kriegsmann K., Streit H., Meliß R.R., Müller C.S.L., Kriegsmann J. (2023). Imaging Mass Spectrometry for the Classification of Melanoma Based on BRAF/NRAS Mutational Status. Int. J. Mol. Sci..

[14] Mansouri A., Varukattu N.B., Curole B.J., Moaven O., Adamec J. (2026). Mass spectrometry imaging tutorial: From cancer biomarker discovery to clinical applications. Anal. Chim. Acta.

[15] Yang C.C., Lin C.Y., Yuan H.Y., Huang H.C., Juan H.F. (2026). Mass spectrometry-based human spatial omics: Fundamentals, innovations, and applications. J. Biomed. Sci..

[16] Piga I., Magni F., Smith A. (2024). The journey towards clinical adoption of MALDI-MS-based imaging proteomics: From current challenges to future expectations. FEBS Lett..

[17] Smith A. (2025). Recent developments in spatial proteomics with MALDI mass spectrometry imaging: A journey toward clinical adoption. Expert Rev. Proteom..

[18] Grgic A., Cuypers E., Dubois L.J., Ellis S.R., Heeren R.M.A. (2024). MALDI MSI Protocol for Spatial Bottom-Up Proteomics at Single-Cell Resolution. J. Proteome Res..

[19] Xing L., Zhao C.-L., Mou H.-Z., Pan J., Kang B., Chen H.-Y., Xu J.-J. (2023). Next Generation of Mass Spectrometry Imaging: From Micrometer to Subcellular Resolution. Chem. Biomed. Imaging.

[20] Casadonte R., Kriegsmann J., Kriegsmann M., Kriegsmann K., Torcasio R., Gallo Cantafio M.E., Viglietto G., Amodio N. (2023). A Comparison of Different Sample Processing Protocols for MALDI Imaging Mass Spectrometry Analysis of Formalin-Fixed Multiple Myeloma Cells. Cancers.

[21] Zhu X., Xu T., Peng C., Wu S. (2022). Advances in MALDI Mass Spectrometry Imaging Single Cell and Tissues. Front. Chem..

[22] Piga I., Pagni F., Magni F., Smith A. (2023). Cytological Cytospin Preparation for the Spatial Proteomics Analysis of Thyroid Nodules Using MALDI-MSI. Methods Mol. Biol..

[23] Boskamp T., Lachmund D., Casadonte R., Hauberg-Lotte L., Kobarg J.H., Kriegsmann J., Maass P. (2020). Using the Chemical Noise Background in MALDI Mass Spectrometry Imaging for Mass Alignment and Calibration. Anal. Chem..

[24] Kong A.T., Leprevost F.V., Avtonomov D.M., Mellacheruvu D., Nesvizhskii A.I. (2017). MSFragger: Ultrafast and comprehensive peptide identification in mass spectrometry-based proteomics. Nat. Methods.

[25] Veličković D., Veličković M., O’Connor C.L., Bitzer M., Anderton C. (2025). The Impact of the Mass Analyzer and Tissue Section Thickness on Spatial N-Glycomics with MALDI-MSI. J. Am. Soc. Mass Spectrom..

[26] Longuespée R., Kriegsmann K., Cremer M., Zgorzelski C., Casadonte R., Kazdal D., Kriegsmann J., Weichert W., Schwamborn K., Fresnais M. (2019). In MALDI-Mass Spectrometry Imaging on Formalin-Fixed Paraffin-Embedded Tissue Specimen Section Thickness Significantly Influences *m*/*z* Peak Intensity. Proteom. Clin. Appl..

[27] Schwamborn K., Caprioli R.M. (2010). MALDI imaging mass spectrometry-painting molecular pictures. Mol. Oncol..

[28] Noye T.M., Mittal P., Price Z.K., Fewster A., Williams G., Pukala T.L., Klingler-Hoffmann M., Hoffmann P., Oehler M.K., Lokman N.A. (2025). Identification of Proteins Associated with Ovarian Cancer Chemotherapy Resistance Using MALDI-MSI. Int. J. Mol. Sci..

[29] Hanahan D., Weinberg R.A. (2011). Hallmarks of cancer: The next generation. Cell.

[30] Valdivia A., Isac A.M., Cardenas H., Zhao G., Zhang Y., Huang H., Wei J.-J., Cuello-Fredes M., Kato S., Gomez-Valenzuela F. (2025). Complement activation at the interface between adipocytes and cancer cells drives tumor progression. JCI Insight.

[31] Cho M.S., Rupaimoole R., Choi H.J., Noh K., Chen J., Hu Q., Sood A.K., Afshar-Kharghan V. (2016). Complement Component 3 Is Regulated by TWIST1 and Mediates Epithelial-Mesenchymal Transition. J. Immunol..

[32] Hoffman L.M., Jensen C.C., Kloeker S., Wang C.L., Yoshigi M., Beckerle M.C. (2006). Genetic ablation of zyxin causes Mena/VASP mislocalization, increased motility, and deficits in actin remodeling. J. Cell Biol..

[33] Kim H.S., Choi J.Y., Jang S.H., Kang M., Yoon M.G., Baek G.O., Park W., Han J.E., Cho H.J., Jeong J.Y. (2025). Fibrinogen alpha and beta chains as non-invasive predictors of hepatocellular carcinoma progression. Sci. Rep..

[34] Battaglia A.M., Sacco A., Vecchio E., Scicchitano S., Petriaggi L., Giorgio E., Bulotta S., Levi S., Faniello C.M., Biamonte F. (2023). Iron affects the sphere-forming ability of ovarian cancer cells in non-adherent culture conditions. Front. Cell Dev. Biol..

[35] Torti S.V., Torti F.M. (2013). Iron and cancer: More ore to be mined. Nat. Rev. Cancer.

[36] Wisastra R., Dekker F.J. (2014). Inflammation, Cancer and Oxidative Lipoxygenase Activity are Intimately Linked. Cancers.

[37] Almeida-Nunes D.L., Nunes M., Osorio H., Ferreira V., Lobo C., Monteiro P., Abreu M.H., Bartosch C., Silvestre R., Dinis-Oliveira R.J. (2024). Ovarian cancer ascites proteomic profile reflects metabolic changes during disease progression. Biochem. Biophys. Rep..

[38] Nieman K.M., Kenny H.A., Penicka C.V., Ladanyi A., Buell-Gutbrod R., Zillhardt M.R., Romero I.L., Carey M.S., Mills G.B., Hotamisligil G.S. (2011). Adipocytes promote ovarian cancer metastasis and provide energy for rapid tumor growth. Nat. Med..

[39] Kahnt A.S., Häfner A.K., Steinhilber D. (2024). The role of human 5-Lipoxygenase (5-LO) in carcinogenesis—A question of canonical and non-canonical functions. Oncogene.

[40] Rudberg P.C., Tholander F., Thunnissen M.M., Samuelsson B., Haeggstrom J.Z. (2002). Leukotriene A4 hydrolase: Selective abrogation of leukotriene B4 formation by mutation of aspartic acid 375. Proc. Natl. Acad. Sci. USA.

[41] Carron J., Coser L.O., Lima C.S.P., Lourenço G.J. (2024). The impact of ERP29 on the progression of pharyngeal squamous cell carcinoma. Sci. Rep..

[42] Zhang D., Richardson D.R. (2011). Endoplasmic reticulum protein 29 (ERp29): An emerging role in cancer. Int. J. Biochem. Cell Biol..

[43] Martincuks A., Li P.C., Zhao Q., Zhang C., Li Y.J., Yu H., Rodriguez-Rodriguez L. (2020). CD44 in Ovarian Cancer Progression and Therapy Resistance—A Critical Role for STAT3. Front. Oncol..

[44] Duan C., Li K., Pan X., Wei Z., Xiao L. (2023). Hsp90 is a potential risk factor for ovarian cancer prognosis: An evidence of a Chinese clinical center. BMC Cancer.

[45] Li C., Hao B., Yang H., Wang K., Fan L., Xiao W. (2024). Protein aggregation and biomolecular condensation in hypoxic environments (Review). Int. J. Mol. Med..

[46] Sharif F., Rasul A., Ashraf A., Hussain G., Younis T., Sarfraz I., Chaudhry M.A., Bukhari S.A., Ji X.Y., Selamoglu Z. (2019). Phosphoglycerate mutase 1 in cancer: A promising target for diagnosis and therapy. IUBMB Life.

[47] Panupinthu N., Yu S., Zhang D., Zhang F., Gagea M., Lu Y., Grandis J.R., Dunn S.E., Lee H.Y., Mills G.B. (2014). Self-reinforcing loop of amphiregulin and Y-box binding protein-1 contributes to poor outcomes in ovarian cancer. Oncogene.

